# Effect of limb ischemic preconditioning on myocardial apoptosis-related proteins in ischemia-reperfusion injury

**DOI:** 10.3892/etm.2013.977

**Published:** 2013-02-26

**Authors:** JIANZHI GAO, LINJING ZHAO, YONGLING WANG, QINGLEI TENG, LIDONG LIANG, JINYING ZHANG

**Affiliations:** 1 School of Basic Medical Sciences, Xinxiang Medical University, Xinxiang, Henan 453003;; 2 Department of Anesthesia, Third Affiliated Hospital of Xinxiang Medical University, Xinxiang, Henan 453003;; 3 Department of Cardiology, First Affiliated Hospital of Zhengzhou University, Zhengzhou, Henan 450000, P.R. China

**Keywords:** limb ischemic preconditioning, myocardium, ischemiareperfusion injury, B cell lymphoma 2, caspase-3, apoptosis

## Abstract

The aim of this study was to investigate the effect of limb ischemic preconditioning (LIPC) on myocardial apoptosis in myocardial ischemia-reperfusion injury (MIRI), as well as the regulation of caspase-3 and the B cell lymphoma 2 (Bcl-2) gene in LIPC. A total of 50 rats were divided randomly into 5 groups (n=10). Four rats in each group were drawn out for detection of apoptosis. The sham, MIRI and LIPC groups underwent surgery without additional treatment. In the LY294002 group, LY294002 preconditioning was administered 15 min before reperfusion. In the LY294002+LIPC group, following LIPC, LY294002 was administered 15 min before reperfusion. The relative expression of myocardial Bcl-2 and caspase-3 mRNA and the apoptotic index for each group were determined by reverse transcription-polymerase chain reaction (RT-PCR) and terminal deoxynucleotidyl transferase deoxyuridine triphosphate (dUTP) nick end labeling (TUNEL), respectively. The ultrastructure of the cardiac muscle tissues was observed by election microscopy. Compared with the sham group, the expression of caspase-3 mRNA in the MIRI group significantly increased (P<0.05) and the expression of Bcl-2 mRNA clearly decreased. Compared with the MIRI group, LIPC reduced the expression of caspase-3 and increased the expression of Bcl-2 mRNA (P<0.05). There were no significant differences between the LY294002+LIPC group and the MIRI group. Compared with the sham group, the apoptotic index of myocardial cells in the MIRI group significantly increased (P<0.05). Compared with the MIRI group, LIPC significantly decreased the apoptotic index of myocardial cells (P<0.05) and LY294002 increased the apoptotic index of myocardial cells. Compared with the LIPC group, LY294002+LIPC significantly increased the apoptotic index of myocardial cells (P<0.05). There were no significant differences between the LY294002+LIPC and MIRI groups. In conclusion, LIPC increased the expression of Bcl-2 and decreased caspase-3 mRNA and apoptosis in myocardial tissue following MIRI. Therefore, LIPC plays a protective role in myocardial tissue.

## Introduction

The pathogenesis of myocardial ischemia-reperfusion injury (MIRI) is complex and the mechanisms involved have not yet been fully elucidated. In previous years, basic and clinical research of molecular cardiology have demonstrated that apoptosis occurs in a number of physiological and pathological processes of the cardiovascular system (1). Studies have shown that apoptosis may be one of the pathogenetic processes of reperfusion injury. The possibility of reducing apoptosis and the severity of reperfusion injury through interfering with the expression of apoptosis-related genes has received considerable attention. Therefore, determining how to reduce myocardial apoptosis during the MIRI process has become one of the hot topics in the field of myocardial protection (2,3).

Apoptosis is an inevitable phenomenon and plays an important role in biological development and the maintenance of biological balance. It is capable of clearing unnecessary cells, thus promoting normal body development. When an individual matures, apoptosis is a mechanism of physiological regulation and self-protection (4), through which injured cells are removed to maintain the regeneration of normal cells. Under physiological conditions, apoptosis is the main cause of cell death and under pathological conditions, abnormal apoptosis may lead to a variety of diseases (5). Myocardial apoptosis involves a variety of genes and proteins, which are controlled by the anti-apoptosis promoting signal transduction pathway. A deficiency in the myocardial cell survival factor means the signal transduction pathway may not be able to be activated or myocardial cells undergo apoptosis under the stimulation of apoptotic factors (6).

This study aimed to observe the effects of limb ischemic preconditioning (LIPC) on myocardial apoptosis-related protein in MIRI with the specific phosphoinositide 3-kinase (PI3k) inhibitor LY294002 *in vivo*.

## Materials and methods

### Materials

A terminal deoxynucleotidyl transferase deoxyuridine triphosphate (dUTP) nick end labeling (TUNEL) kit and reverse transcription-polymerase chain reaction (RT-PCR) kit were purchased from Beijing Zhongshan Golden Bridge Biotechnology Co., Ltd. (Beijing, China). Primers for amplifying β-actin, caspase-3 and B cell lymphoma 2 (Bcl-2) were synthesized by Beijing Sanbo Yuanzhi Biotechnology Co., Ltd. (Beijing, China). DNA markers and TRIzol reagent were also used.

### Experimental groups and treatment

A total of 50 Sprague-Dawley (SD) male rats, weighing 220–280 g, were provided by the Experimental Animal Center of Henan Province. The rats were divided into five groups (n=10 in each group): i) sham surgery group: the surgical procedure was similar to that of the MIRI group, with the exception that the left anterior descending coronary artery was strung without ligation; ii) MIRI group: the left anterior descending coronary artery was ligated for 30 min, followed by perfusion for 120 min; iii) LIPC group: the femoral artery was continuously blocked at the upper 1/3 section for 5 min, followed by continuous reperfusion for 5 min and this process was repeated 3 times. The ischemia-reperfusion of the femoral artery was implemented for 3 days and MIRI was performed on the 4th day with the same surgical procedure as in the MIRI group. iv) LY294002 pretreatment group: the rats were pretreated with LY294002 following myocardial ischemia and 15 min before reperfusion (injection dose into the femoral vein, 0.3 *μ*g/g body weight) and v) LY294002+LIPC group: rats were pretreated with LY294002 following LIPC and 15 min before reperfusion. Following the treatment of each group, myocardial tissue was immediately removed from the ischemic center of the hearts of the rats. One portion of the tissue was placed in sterile pyrogen-free vials and stored at −78°C and the other portion was fixed with 4% paraformaldehyde. This study was approved by the ethics committee of Xinxiang Medical University.

### RT-PCR detection

TRIzol reagent was used to extract total RNA from the myocardial tissue. RT-PCR was performed by two steps: i) a total of 12 *μ*g total RNA from the myocardial tissue was used to prepare 20 *μ*l for the reverse transcription system and the RNA was reverse transcribed to cDNA using oligo (dT) 15 primer and AMV reverse transcriptase at 72°C for 10 min, 42°C for 1 h and 70°C for 10 min. ii) The cDNA was used as a template to amplify adenosine diphosphate (ADP) and ADP receptor 1 (ADPR1) with PCR amplification of β-actin as an internal control. β-actin: 379 bp, forward primer: 5′-CAGTAACAGTCCGCCTAGAA-3′, reverse primer: 5′-GATTACTGCTCTGGCTCCTA-3′ (94°C for 45 sec, 58°C for 45 sec and 72°C for 45 sec, 35 cycles). Bcl-2, 315 bp, forward primer: 5′-CCGCTACCGCCGCGACTTC-3′, reverse primer: 5′-AAACAGAGGCCGCATGCTG-3′. Caspase-3, 279 bp, forward primer: 5′-TGTCATCTCGCTCTGGTACG-3′, reverse primer: 5′-AAATGACCCCTTCATCACCA-3′. PCR products were observed by ionization on a 1.5% agarose gel and imaged.

### In situ TUNEL assay

Experiments were performed according to the manufacturer’s instructions. Cells were stained with diaminobenzidine (DAB), counterstained with hematoxylin, dehydrated with gradient alcohol, cleared with xylene and mounted with neutral resin. The reaction mixture was replaced with phosphate-buffered saline (PBS) in the negative control. The nucleus was blue and the apoptotic nucleus was brownish-black or brown. Four slices from each rat were observed and five fields (magnification, ×400) on each slice were counted. The percentage of positive apoptotic nuclei in the total number of cells per field was calculated and the mean value was the apoptotic index of the myocardial cells.

### Transmission electron microscopy (TEM)

Myocardial tissues were fixed in 2.5% glutaraldehyde in PBS and the ultra-microstructure and morphology of the myocardial tissue were observed and imaged using TEM.

### Statistical analysis

SPSS v13.0 (SPSS Inc., Chicago, IL, USA) was used to perform statistical analysis. Quantitative data were expressed as mean ± standard deviation. Single factor analysis of variance was performed to analyze the differences between the groups. P<0.05 was considered to indicate a statistically significant difference.

## Results

### Detection of apoptosis by TUNEL assay

**Figure 1:**
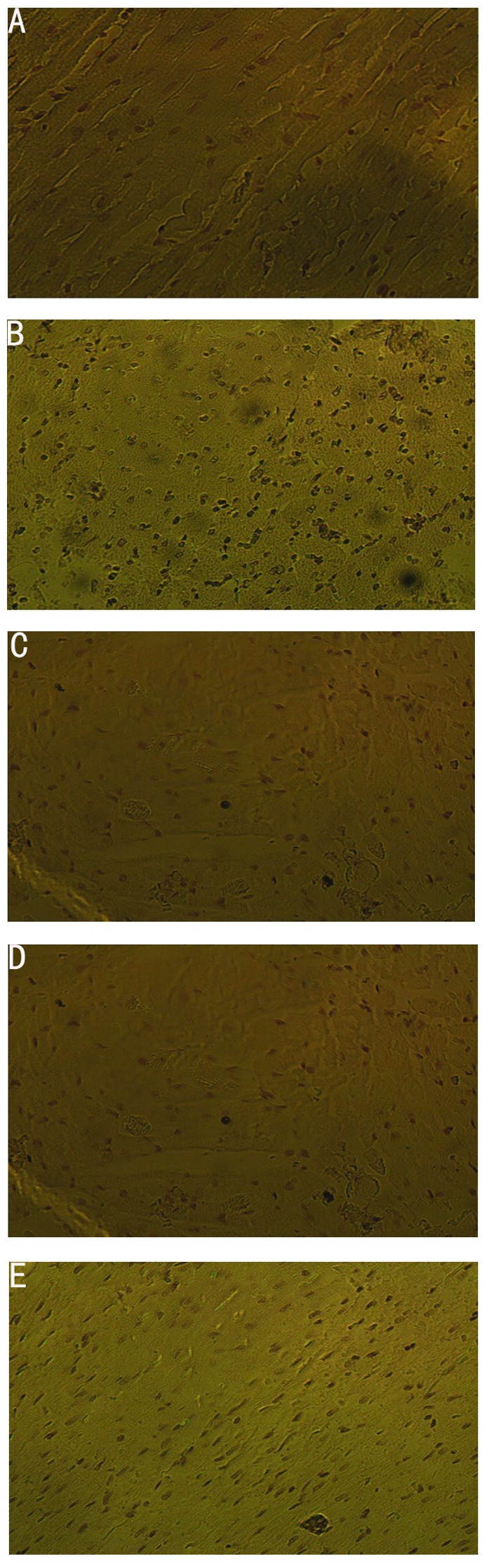
Comparison of cardiomyocyte apoptosis in each group (magnification, ×400). (A) Sham group; (B) MIRI group; (C) LIPC group; (D) LY294002 group and (E) LY294002+LIPC group. MIRI, myocardial ischemia-reperfusion injury; LIPC, limb ischemic preconditioning.

A brown nucleus represented the occurrence of apoptosis and the apoptotic index (AI) was calculated. The results were as follows: apoptosis of myocardial cells in the sham group was rare; the number of positive apoptotic cells in the MIRI group increased significantly compared to the sham group (P<0.05); the number of positive apoptotic cells in the LIPC group decreased significantly compared to that of the MIRI group (P<0.05); the number of apoptotic cells in the LY294002 group increased significantly and the number of apoptotic cells in the LY290024+LIPC group increased significantly compared to that of the LIPC group (P<0.05), but was not significantly different from that of the MIRI group (Table I). These results indicate that LIPC is capable of reducing MIRI-induced myocardial apoptosis through the ADP/PI3k/Akt signaling pathway (Fig. 1).

### Changes of myocardial ultra-micro structure under TEM

**Figure 2:**
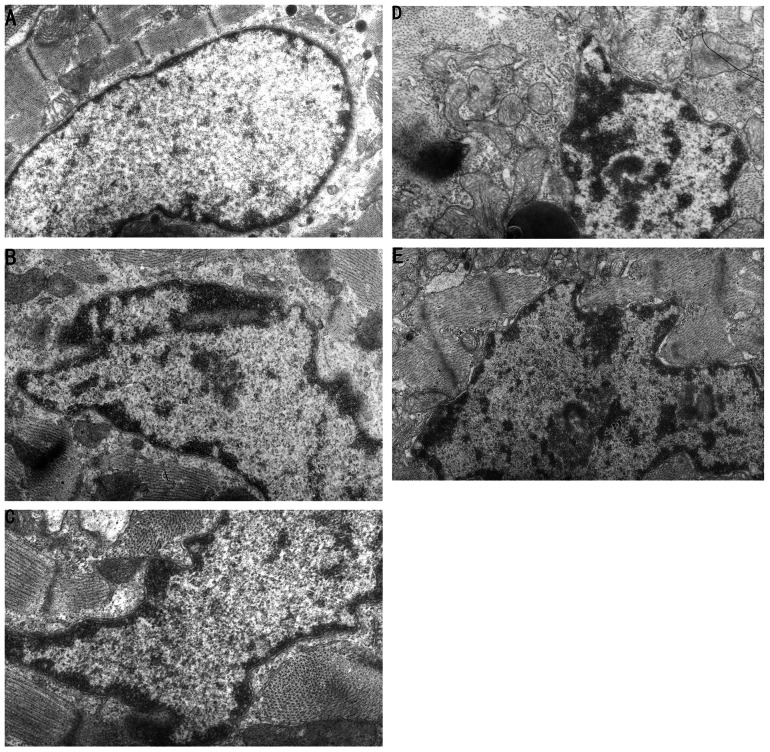
Ultrastructural changes of cardiomyocytes in each group under transmission electron microscopy (magnification, ×10^4^). (A) Sham group; (B) MIRI group; (C) LIPC group; (D) LY294002 group and (E) LY294002+LIPC group. MIRI, myocardial ischemia-reperfusion injury; LIPC, limb ischemic preconditioning.

TEM revealed normal cell structure, a dense arrangement of myocardial perinuclear myofilaments and developed intracytoplasmic mitochondria in the sham group. In the MIRI, LY294002 and LY294002+LIPC groups, TEM revealed damaged myocardial cell structure, expanded rough endoplasmic reticulum, swollen mitochondria, faded matrix and unclear crest and increased heterochromatin with margination, with an appearance of degenerated and necrotized myocardial cells. In the LIPC group, TEM revealed mild edema of the myocardial cells, mild disordered arrangement of perinuclear myofilaments, no evident swelling of mitochondria and increased heterochromatin without clear margination. The MIRI group treated with LY294002 and LIPC had significantly reduced heterochromatin and margination compared to the group treated with only LY294002 (Fig. 2).

### Expression of apoptotic factors, caspase-3 and Bcl-2, in each group

**Figure 3:**
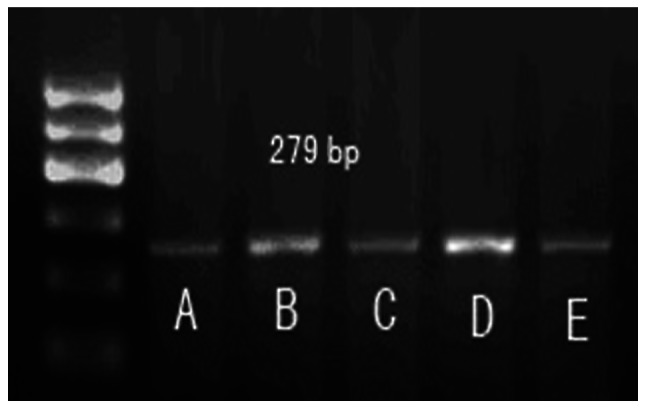
Expression of caspase-3 mRNA in the myocardial tissue of each group. (A) Sham group; (B) MIRI group; (C) LIPC group; (D) LY294002 group and (E) LY294002+LIPC group. MIRI, myocardial ischemia-reperfusion injury; LIPC, limb ischemic preconditioning.

**Figure 4:**
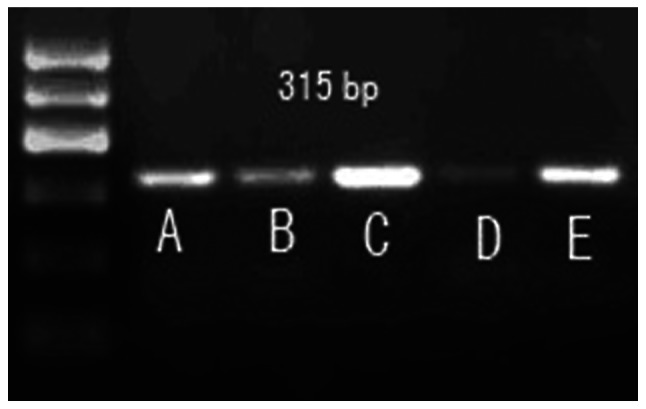
Expression of Bcl-2 mRNA in the myocardial tissue of each group. (A) Sham group; (B) MIRI group; (C) LIPC group; (D) LY294002 group and (E) LY294002+LIPC group. Bcl-2, B cell lymphoma 2; MIRI, myocardial ischemia-reperfusion injury; LIPC, limb ischemic preconditioning.

**Figure 5:**
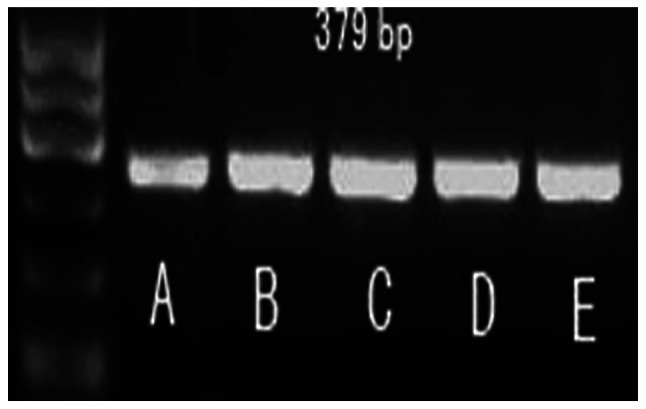
Expression of β-actin mRNA in the myocardial tissue of each group. (A) Sham group; (B) MIRI group; (C) LIPC group; (D) LY294002 group and (E) LY294002+LIPC group. MIRI, myocardial ischemia-reperfusion injury; LIPC, limb ischemic preconditioning.

Compared to the sham group, the expression level of caspase-3 mRNA in the MIRI group significantly increased (P<0.05); however, the expression of Bcl-2 mRNA reduced significantly. Compared to the MIRI group, LIPC decreased the caspase-3 mRNA expression level (P<0.05) and increased the Bcl-2 mRNA expression level (P<0.05), while LY294002 increased the caspase-3 mRNA expression level (P>0.05) and reduced the Bcl-2 mRNA expression level (P>0.05). There was no significant difference in mRNA expression levels of caspase-3 and Bcl-2 between the LY294002+LIPC and MIRI groups. These results indicate that LY294002 is capable of reducing the protection effects of LIPC on the MIRI cardiac muscles of rats by changing the mRNA expression levels of caspase-3 and Bcl-2 (Table II, Figs. 3–5).

## Discussion

A previous study identified that apoptosis plays an important role in MIRI. Reperfusion injury leads to cell death through necrosis and apoptosis (7). Laboratory and clinical data have indicated that, due to the fact that myocardial apoptosis is relatively severe during the reperfusion period, it contributes greatly to myocardial infarction and ventricular remodeling (8). Apoptosis is programmed cell death and is also the final result of the cell death signal regulation by stimulating factors. Currently, the most commonly accepted theory is that apoptosis is co-regulated by pro-apoptotic genes, which detect signals from pro-apoptotic factors through signal transduction pathways and anti-apoptotic genes.

The mechanisms of MIRI-induced apoptosis are complex and the details of the process are not entirely clear. However, one study demonstrated that the myocardial infarction area is the location of the integration of apoptosis and necrosis. Zhao *et al*(9) identified that the number of apoptotic cells in the surrounding tissue of the infarcted myocardium is directly proportional to the reperfusion time, which further proves the effect of apoptosis on the myocardial infarction area. It has been proposed that apoptosis is the major mode of cell death for early myocardial infarction. In an animal study, decreasing the number of apoptotic cells decreased the infarct area and improved the systolic function of the heart (4). Therefore, inhibiting apoptosis may be used as an effective measure in treating acute myocardial infarction.

In the present study, LIPC significantly inhibited reperfusion injury-induced apoptosis. The results demonstrated that apoptosis was rare in the sham group and cardiomyocyte apoptosis in the MIRI group increased. The number of apoptotic cells in the LIPC group was significantly reduced in comparison to that of the MIRI group and the number of apoptotic cells in the LY294002-pretreatment group was the largest and was significantly different from the other groups. These results suggest that apoptosis plays an important role in MIRI.

LIPC inhibited apoptosis; however, the number of apoptotic cells in the LY294002-pretreatment group was the largest and the number of myocardial apoptotic cells in the LY294002+LIPC co-treatment group was significantly reduced compared to that in the LY294002-pretreated group. This further indicates that LIPC may inhibit cardiomyocyte apoptosis through the ADP/PI3k/Akt signal pathway. Under TEM, we observed that in the MIRI group, myocardial cell structure was damaged with degeneration and necrosis of myocardial cells. In the LIPC group, myocardial cells presented mild edema with mild disordered arrangement of myofilaments surrounding the nuclei. In the LY294002-pretreatment group, myocardial cell structural damage was evident, with a large amount of degenerated and necrotized myocardial cells. We observed that cell damage in MIRI rats following co-treatment with LY294002 and LIPC was clearly reduced compared to the group treated with LY294002 only. This is consistent with the results discussed above.

Apoptosis, one of the main pathological processes induced by MIRI, involves a variety of cellular signal transduction pathways. The Bcl-2 family is a group of important apoptosis-regulating proteins that are expressed on the mitochondrial outer membrane, endoplasmic reticulum membrane and nuclear membrane. Overexpression of Bcl-2 proteins blocks the pro-apoptosis signal transduction pathway, thereby inhibiting the activation of caspase-3 and ultimately preventing apoptosis caused by the caspase cascade (10).

Inhibition of apoptosis by Bcl-2 may be related to the following mechanisms: i) overexpression of Bcl-2 may decrease the production of oxygen radicals and the formation of lipid peroxides; ii) overexpression of Bcl-2 prevents the increase of mitochondrial permeability and reduces the release of proapoptotic proteins, thus inhibiting apoptosis; iii) Bcl-2 may inhibit the Ca^2+^ transmembrane flow and regulate apoptosis by regulating intracellular Ca^2+^ concentration and iv) Bcl-2 may bind to the apoptotic protease to achieve its anti-apoptotic effects (11).

It has been suggested that members of the caspase family are the key factors involved in apoptosis and caspase activation and overexpression induces apoptosis; therefore, they are also known as apoptotic proteases. When caspases are translated, they exist in the cytoplasm in the form of inactive zymogen. When the cells are stimulated by external physiological or pathological factors, apoptosis is initiated and the caspases are activated through a series of cleavage cascading reactions. Due to cascade enlarging effects and positive feedback, a large number of proteases are activated instantly, which causes simultaneous and rapid decomposition of a variety of ‘cell death substrates’, eventually leading to chromosome breakage, morphological changes of cells and finally apoptosis (12).

Caspase-3 is an important member of the cysteine protease family and one of the most important regulating genes in the apoptotic pathway. It digests cell structure proteins and directly causes cell apoptosis. Caspase-3 plays an important role in apoptosis and is considered to be an apoptosis marker enzyme (13). When the body is stimulated by internal or external factors, caspase-3 zymogen is cleaved by various proteases and then activated. Activation of caspase-3 leads to apoptosis of a variety of cells and a number of apoptosis-triggering factors ultimately induce apoptosis through caspase-3-mediated signal transduction pathways. A previous study reported that caspase-3 is involved in the genesis and development of MIRI and the formation of apoptotic bodies (14).

In this study, RT-PCR was used to detect the effects of LIPC on the mRNA expression level of caspase-3 and Bcl-2 in myocardial cells. The results demonstrated that LIPC decreased the mRNA expression level of caspase-3 and increased the mRNA expression level of Bcl-2. However, LY290024 increased the mRNA expression level of caspase-3 and decreased the expression level of Bcl-2 mRNA, which was not significantly different from the results of the MIRI group. Co-treating MIRI rats with LY294002 and LIPC resulted in significant differences in mRNA expression levels of caspase-3 and Bcl-2 from the LY294002-only group.

These results indicate that myocardial ischemia-reperfusion-induced cardiac apoptosis is a result of increased caspase-3 expression levels and decreased Bcl-2 expression levels. LIPC may reverse the above phenomenon, which demonstrates that LIPC may play a role in protecting myocardial cells by increasing the expression level of anti-apoptotic protein, Bcl-2 and reducing the expression level of pro-apoptotic protein, caspase-3. The present study demonstrated that at the molecular level the expression level of apoptosis-related proteins in rat myocardium following LIPC and before reperfusion may be changed significantly; therefore, enhancing the ability of LIPC activates the cardiac survival signaling pathway.

## Figures and Tables

**Table I:** Comparison of the index of cardiomyocyte apoptosis of each group (mean ± SD, n=4).

Group	Apoptotic index (%)
Sham	2.01±0.44^a,b,c^
MIRI	18.68±2.81^b,c,d^
LIPC	8.17±1.93^a,c,d,e^
LY294002	23.76±3.77^a,b,d,e^
LY294002+LIPC	16.10±2.06^b,c,d^

a P<0.01 vs. MIRI,

b P<0.01 vs. LIPC,

c P<0.01 vs. LY294002,

d P<0.01 vs. sham,

e P<0.01 vs. LY294002+ LIPC.

**Table II:** Relative levels of caspase-3 and bcl-2 mRNA/β-actin in the myocardial tissue of each group (mean ± SD, n=6).

Group	Caspase-3/β-actin	bcl-2/β-actin
Sham	0.31±0.05^a,b,c^	0.51±0.04^a,b,c,d^
MIRI	0.62±0.09^d,e^	0.31±0.05^d,e^
LIPC	0.35±0.07^a,c,d^	0.74±0.12^a,b,c,e^
LY294002	0.68±0.13^c,d,e^	0.24±0.03^c,d,e^
LY294002+LIPC	0.56±0.11^b,d,e^	0.32±0.04^b,d,e^

a P<0.01 vs. MIRI,

b P<0.01 vs. LY294002;

c P<0.01 vs. LY294002+LIPC,

d P<0.01 vs. LIPC,

e P<0.01 vs. sham.
